# Emerging roles of transfer RNA fragments in the CNS

**DOI:** 10.1093/brain/awaf130

**Published:** 2025-04-28

**Authors:** Katarzyna Winek, Hermona Soreq

**Affiliations:** Leibniz Institute on Aging, Fritz Lipmann Institute (FLI), Jena 07745, Germany; The Edmond and Lily Safra Center of Brain Science, The Hebrew University of Jerusalem, Jerusalem 9190401, Israel; The Silberman Institute of Life Sciences, The Hebrew University of Jerusalem, Jerusalem 9190401, Israel

**Keywords:** brain ageing, tRNA-derived small RNAs (tsRNAs), neurodegeneration, neuroprotection, sex differences, tRNA mutations

## Abstract

Transfer RNA-derived small RNAs (tsRNAs), previously considered inactive tRNA degradation products, have now been shown to be functional small non-coding RNAs. They may play important roles within the CNS and in brain-body interactions, both during normal developmental stages as well as in diverse brain pathologies. Among the cell types found in the CNS, tsRNAs are particularly abundant in neurons. Correspondingly, neurons show cell type specific tRNA expression profiles when compared to other cells of the CNS under homeostatic conditions and defects in tRNA processing may lead to neurological disorders. Disease-specific tsRNA profiles have been identified in a number of CNS disorders, including amyotrophic lateral sclerosis and epilepsy. Elevated levels of specific tsRNAs have been found in the blood before the onset of epileptic seizures; and age-related, sex-specific loss of mitochondrial genome-originated tsRNAs in the nucleus accumbens of female patients is correlated with accelerated cognitive deterioration in Alzheimer's disease. Disease-related tsRNA signatures have also been identified in the CSF of patients with Parkinson's disease, and nucleated blood cells from ischaemic stroke patients show specific elevation of cholinergic-targeted tsRNAs. The mechanisms of action of tsRNAs are still being elucidated but include targeting complementary mRNA to impact RNA levels and translation in a miRNA-like manner, direct interaction with RNA binding proteins, or interference with translation machinery. The function of tsRNAs may be affected by the chemical modifications they inherit from the originating tRNA molecules, which impact tsRNAs production and may modulate their interactions with proteins. Research on the genetics, biochemical properties and regulatory roles of tsRNAs has expanded rapidly in recent years, facilitated by novel sequencing strategies, which include the removal of tRNA modifications and chemically blocked ends that hinder amplification and adapter ligation. Future in-depth profiling of tsRNAs levels, mode(s) of function, and identification of interacting proteins and RNAs may together shed light on the impact of tsRNAs on neuronal function, and enable novel diagnostics/therapeutics avenues for brain diseases in age, sex and disease-specific manner.

## Teaser

Once thought to be inactive transfer RNA (tRNA) degradation products, tRNA-derived small RNAs (tsRNAs) are now recognized as functional small non-coding RNAs. Winek and Soreq review their roles in the CNS, highlighting their contribution to maintaining brain health and their involvement in disease processes.

## tsRNAs re-emerge as functional molecules

Small non-coding RNAs play important roles as regulators of transcription and translation, providing an additional layer of control over these processes.^1^ Transfer RNA fragments (further termed tsRNAs, tRNA-derived small RNAs) are cleavage products of tRNAs and their precursors, which were recently rediscovered as an intriguing novel family of functionally active small non-coding RNAs. In all life forms, the role of tRNAs is to transport amino acids to the translational machinery. While tsRNA sequences can be identified in small RNA-sequencing datasets, until recently they were largely considered to be inactive degradation products of tRNAs. In the past decades, tsRNAs have been shown to actively modulate cellular processes at multiple levels. Many unknowns remain, however, regarding tsRNAs function and mode of action, and this novel family of small non-coding RNAs may play a role in normal physiology as well as in disease pathogenesis. Here, we take a neurocentric perspective on tsRNAs, and will review recent advances in understanding their roles in normal CNS function as well as in neurological disease. We discuss the role of these newly described regulatory RNAs, which may operate in the CNS and explore future directions of brain tsRNAs research.

### The basics of tsRNAs: a beginner's guide

tsRNAs may be derived from tRNAs encoded in either the nuclear or mitochondrial genome, which together harbour >600 tRNA genes in *Homo sapiens*.^2^ However, only some of those genes are actively transcribed, and the inactive ones may play a role in supporting genome structure.^3^ tsRNAs are derived from mature tRNA molecules or their precursors, and excessive tsRNAs production might significantly deplete specific tRNA levels, interrupting their crucial activities in protein translation. Indeed, reduced tRNA-Tyr pools due to tRNA fragmentation were shown to impair cellular growth under oxidative stress.^4^ Yet, in most cases, stress-induced tRNA cleavage in eukaryotic cells does not have a drastic effect on the parent tRNAs available for translation^5-8^ as only ∼1% of parent tRNA is cleaved into fragments.^9^ tRNA isodecoders (tRNAs having the same anticodon but differing in the sequence of the tRNA molecule) gene expression differences identified in the human brain and HEK293T cells^10^ may also be a source of tsRNA diversity, as isodecoder tRNAs are redundant in translation. It has been argued that some tRNA transcripts might be solely produced as a resource for generating tsRNAs.^10^

**Figure 1:**
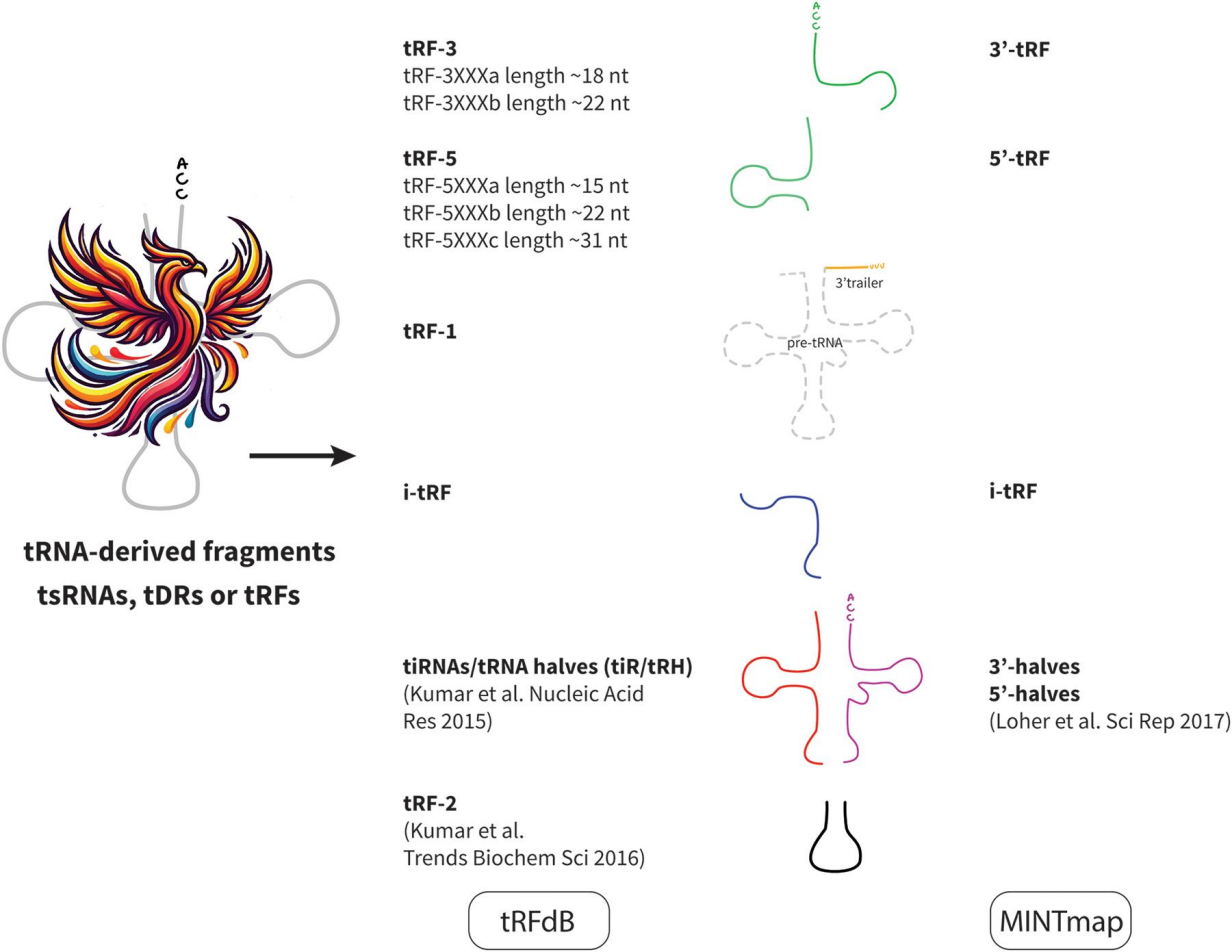
**Several classification systems of tRNA-derived fragments have been suggested to date and a unified version is urgently needed**. Here, we provide an example of two popular classifications (tRFdb, on the *left* and MINTmap, on the *right*).^17-19^ The Phoenix icon was generated by an artificial intelligence tool, ChatGPT image generator https://chatgpt.com/g/g-pmuQfob8d-image-generator (with modifications). tDR = tRNA-derived RNA; tRF = tRNA-derived fragment; tsRNA = tRNA-derived small RNAs.

Multiple cleavage sites in the tRNA molecule, combined with nuclease activities, lead to production of distinct tsRNA types and lengths. In human cells, the nuclease angiogenin mediates tRNA cleavage at the anticodon loop.^11,12^ This leads to generation of tRNA halves, whose level is elevated under cellular stress^5^ and in hormone-dependent cancers.^13^ Consequently freed 3′- and 5′-tRNA halves can form nicked structures that gain stability in body fluids.^14,15^ Other fragments, derived from the 3′-, 5′- ends or from the internal part of the tRNA—‘i-tRFs’—are produced by Dicer, angiogenin, RNase L and other—as yet—non-identified nucleases, whose actions determine the cleavage site in the tRNA molecule^16^ (Fig. 1). Surprisingly, shorter tsRNAs seemingly do not originate from further fragmentation of the halves, but rather are generated from full-length tRNAs.^12^ Furthermore, at least some of the 3′-tsRNAs are amino-acylated and their levels partially depend on the levels of enzymes catalysing the tRNA charging reaction,^20^ meaning that they were exclusively produced from mature tRNA loaded with an amino acid.

Recent studies revealed a plethora of conditions in which tRNA-derived fragments are involved. Those include numerous cancers,^21-23^ maintenance of sperm viability,^24^ but also paternal transgenerational inheritance^25,26^ and infection.^27-29^ tsRNAs are a widely expressed family of small non-coding RNAs, present in multiple tissues, and implicated in specific processes. As the brain harbours a highly heterogenous population of cells, with an estimated 1:1 glia:neuron ratio,^30^ its collection of tsRNAs is likewise very rich^31^ with many tsRNAs present in a cell-type specific manner, especially in neurons^32^ (refer to the ‘Transfer RNA fragments in the healthy and diseased brain’ section).

Highlighting the novelty of tsRNAs studies, several differing nomenclatures have been developed for these molecules, and the research community still awaits a consensus.^33^ These fragments have been named: (i) tRFs (tRNA-derived fragments, comprising short 3′- and 5′- fragments, tRNA-halves and i-tRFs or alternatively including only tRF-3, tRF-5, and a group of tRF-1, originating from the pre-tRNA and tRF-2 generated from the anticodon loop)^34^; (ii) tsRNAs/tDRs (tRNA-derived small RNAs/tRNA-derived RNAs), further comprising short 3′- and 5′ fragments, i-tRFs, tiRNAs (tRNA-derived stress-induced RNA corresponding to tRNA-halves) and tRNA-halves; or simply (iii) tRFs comprising just 3′-5′-tRFs, i-tRFs and tRNA-halves^17^ (see Fig. 1 for two examples of tsRNAs classification systems). Moreover, nomenclature used for specific molecules may be based on: (i) the length and sequence, generating a specific code for a molecule, such as the one used by the tsRNAs alignment tool MINTmap^17^; (ii) the type of the molecule^18^; or (iii) the parent tRNA and the location of the cleaved fragment.^33^ For example, a fragment derived from the 3′-end of the leucine-carrying tRNA with a sequence ‘ATCCCACCGCTGCCACCA’ would be called ‘tRF-18-HR0VX6D2’, ‘tRF-3001a’ or ‘tDR-59:76-Leu-AAG-1-M6’ depending on the system used. Here, we use the term ‘tsRNAs’ for the entire family of molecules derived from tRNA, further specifying the subclass, if needed.

The emerging new view of the products of tRNA fragmentation see tsRNAs as functional non-random cleavage products, with important physiological functions. In particular, nervous system tsRNAs may be viewed as regulators of cellular processes with potentially altered roles across time, sex and age and in CNS health and disease.^35-39^ Together, tsRNAs may exert comparable and possibly even more impactful effects than those induced by a previously discovered family of small non-coding RNAs, the microRNAs (miRNAs).^40^

## The good, the bad and the ugly: a neurocentric perspective of tsRNAs functions

Combined system neuroscience and cellular biology studies have enabled a better understanding of the role of tsRNAs in the CNS, highlighting both potential neuroprotective and neurotoxic roles of this novel small non-coding RNA family. Those roles may include a function for tsRNAs in keeping the nervous system in homeostatic steady state conditions but also in disease development and progression, or inversely in assisting its recovery from disease states.

### Finding shelter in stress granules

Perhaps the first hint of the neuroprotective role of particular tsRNAs might be the finding that stress-responsive tsRNAs subtypes may accumulate in neuronal stress granules,^41,42^ which form under diverse stress stimuli and keep potentially translatable mRNAs out of the cellular domains involved in routine translation. This saves energy and ensures protective maintenance, for example in vertebrate embryos.^43^ There is still, however, little experimental evidence showing the contribution of tsRNAs to neuronal stress granules formation. The effects of G-quartet-carrying tsRNAs, such as the stress-induced tRNA-Ala halves found in motor neurons,^44^ may provide an example for the neuroprotective function of such tsRNAs. The angiogenin-dependent structure-based capacity of those tsRNAs to organize stress granules may prevent cellular death upon exposure to acute cellular stressors.^44^ Another report highlighted the neuroprotective role of the same angiogenin-cleaved tsRNA-Ala and supported its involvement in stress granule assembly in cells carrying a progranulin (PGRN) mutation.^45^ Stress granules-related protective functions might be particularly relevant in the CNS, where lost neurons are not replaced, and neuronal death is a hallmark of a number of neurodegenerative diseases including amyotrophic lateral sclerosis (ALS), motor neuron demise or fronto-temporal dementia (FTD).^46^ Further support for this idea is the fact that the nuclease angiogenin, which can generate tsRNAs, is neuroprotective in cell culture and mouse neurodegenerative models.^47^ An alternative mechanism of action, which may contribute to the neuroprotective functions of angiogenin, may be activation of the astrocytic nuclear erythroid 2-related factor (Nrf-2), which regulates the response to oxidative stress.^48^ Inversely, some patients with ALS and Parkinson’s disease (PD) carry mutations in the angiogenin gene.^49^

### Harmful fragmentation?

Despite the general benefits of stress granules, their excess and persistence may harm nerve cells and endanger their survival.^50^ Intriguingly, a recent paper showed a correlation of oxidative stress-induced tsRNAs and cell death, and challenged their roles in assembly of stress granules.^51^ In addition, that report highlights problems with cellular stress models used to investigate tRNA fragmentation. Most of the studies in this field focused only on the acute cellular responses and commonly used stress stimuli negatively impact cell viability in the long term, leading to increased release of tsRNAs from dying cells. These tsRNAs, stable in the cell culture medium, may still be signalling molecules with specific functions or have no activity at all.^51^ Another study using neuronal cells, did not identify a protective role of angiogenin but confirmed angiogenin-derived tsRNAs production under several stress conditions, including oxygen and glucose deprivation. The authors argued that the angiogenin and tsRNAs effects may depend on the timing and the stress model used and that harsh stressors leading to rapid cell death may limit the protective potential of tsRNAs^52^; these critiques should be taken into consideration in future studies. As further support for the idea that tRNA fragmentation is deleterious, it has been shown that T cells depend on SLFN2-mediated (schlafen 2) protection of tRNAs from stress-induced cleavage.^53^

Additional evidence of the potentially harmful effects of tsRNAs is that the putamen of patients with Huntington's disease is enriched in tsRNAs. Small RNAs isolated from these specimens, when injected into the striatum of healthy mice, induce motor symptoms characteristic for the disorder.^54^ In addition, loss of RNA processing enzymes in patients with another CNS disorder, pontocerebellar hypoplasia, may lead to the accumulation of specific tsRNAs and subsequent neuronal death.^55^ Mutations in CLP1 (cleavage factor polyribonucleotide kinase subunit 1) and TSEN components (tRNA splicing endonuclease, formed by subunits TSEN2, TSEN15, TSEN34 and TSEN54)^56^ underlie many forms of pontocerebellar hypoplasia. CLP1 belongs to the family of kinases involved in tRNA splicing and acts in cooperation with the TSEN complex. Correspondingly, patients carrying a R140H mutation in CLP1 suffer from pontocerebellar hypoplasia type 10 with microcephaly, defects in brain structure and axonal peripheral neuropathy.^57^ Fibroblasts collected from these patients accumulated fragments derived from isoleucine pre-tRNA introns.^57^  *In vivo* models of mice carrying the CLP1 R140H mutation similarly show accumulation of isoleucine pre-tRNA introns in fibroblasts as well as neurotoxic 5′-tsRNAs from pre-tRNA-Tyr in brains collected after birth.^58^ In another mouse study, introducing a knock-in kinase-dead CLP1 led to build-up of detrimental tsRNAs derived from pre-tRNA-Tyr containing 5′ leader sequences. Those tsRNAs increased neuronal susceptibility to oxidative stress over p53 activation.^59,60^ A recent report pointed out, however, that defects in pre-mRNA cleavage, resulting from the CLP1 mutation rather than tRNA splicing, may contribute to the pathogenesis of type 10 pontocerebellar hypoplasia,^61^ highlighting again the gaps in our knowledge about tsRNAs and their contribution to disease.

In a rat model of ischaemic brain injury, elevated levels of tRNA-Gly and tRNA-Val tsRNAs produced by angiogenin were predicted to negatively impact endothelial cell functions involved in angiogenesis, which would be disadvantageous in tissue repair processes.^62^ Finally, another example of a detrimental role for tsRNAs is the recent observation of accumulation of mitochondrial tRNA-Glu-derived tsRNAs in ageing neurons. This build-up of tsRNAs impaired translation and structural maintenance of mitochondrial cristae in glutamatergic neurons, which in turn damaged neurotransmission due to disrupted glutamate synthesis that has further been linked to memory dysfunction.^63^ A summary of these findings and further studies of tsRNAs in neurological disorders can be found in Table 1.

**Table 1:** Summary of selected tsRNAs studies important in the context of neurological disorders

tsRNA	Condition	Cell/organ	Expression and function	Reference
tRNA-Ala halves	Stress, PGRN A9D mutation—cell culture	Neurons	Stress granule formation and neuroprotection^a^	Ivanov *et al*.^44^ Li *et al*.^45^
Multiple tsRNAs, tsRNAs from tRNA-Ala	Huntington's disease	Brain, putamen	Induce disease symptoms in mice after brain injection	Creus-Muncunill *et al*.^54^
From pre-tRNA Ile introns	CLP1 R140H mutation (PCH type 10)	Fibroblasts	Accumulation of specific tsRNAs	Karaca *et al*.^57^
From pre-tRNA Ile introns and 5′ tsRNAs from pre-tRNA-Tyr	CLP1 R140H mutation mouse model	Newborn mice—fibroblasts, brains	Accumulation of specific tsRNAs	Morisaki *et al*.^58^
5′-tsRNAs from pre tRNA-Tyr	Kinase-dead CLP1 mice	Neurons	Susceptibility to oxidative stress	Hanada *et al*.^59^
	CLP1 R140H mutation mouse model	Neurons	p53-related cell death, inhibition of PKM2 pathway	Inoue *et al*.^60^
From tRNA-Gly and tRNA-Val	Experimental ischaemic brain injury	Endothelial cells	Inhibition of angiogenesis	Li *et al*.^62^
From mt-tRNA-Glu	Ageing—mouse	Neurons	Impairment of translation and glutamatergic transmission	Li *et al*.^63^
5′-tsRNA from tRNA-Glu	Physiological	Cholinergic synapse, mouse brain	Secreted in synaptic vesicles	Li *et al*.^64^
ANG-produced 5′-tsRNAs	Nsun2 knockout mice	Brain	Accelerated neuronal death, defects in neural stem cells	Blanco *et al*.^65^ Flores *et al*.^66^
5′-tsRNAs from tRNA-Ala, tRNA-Gly and tRNA-Glu	Epilepsy	Blood	Increased prior to seizures	Hogg *et al*.^67^
5′-tsRNAs from tRNA-Val	ALS	Blood	Associated with decreased disease progression	Hogg *et al*.^68^
Mainly 3′-tsRNAs	Ischaemic stroke	Blood	Found in immune compartments, may contribute to regulation of monocytes/macrophages	Winek, Lobentanzer *et al*.^69^
tsRNA profile	Ischaemic stroke	Blood	Associated with outcome	Ishida *et al*.^70^
tsRNA profile	MS	Blood/CSF	Opposite expression patterns in cellular compartments	Zheleznyakova *et al*.^71^
tsRNA profile	PD	Blood/CSF	Sex-specific and disease-associated profiles	Paldor *et al*.^72^ Magee *et al*.^73^
tsRNAs (and sncRNAs)	AD	Blood	Associated with disease	Gutierrez-Tordera *et al*.^74^
tRNA-Pro 5′-tsRNAs	AD	Serum, CSF	Associated with disease	Wu *et al*.^75^
From tRNA-Tyr and tRNA-Arg	AD	Brain, cortex	Decreased levels and specific modifications linked with disease	Zhang *et al*.^76^
mt-tsRNAs predicted to target cholinergic mRNAs	AD	Brain, nucleus accumbens	Decreased with age only in females suffering from AD	Shulman *et al*.^77^
5′-tsRNAs and 3′-tsRNAs	ALS, FTD and PD mouse models	Brain	ALS: upregulation of ANG-derived 5′-tsRNAs, ALS, FTD: 3′-tsRNAs downregulation	Baindoor *et al*.^78^

AD = Alzheimer’s disease; ANG = angiogenin; ALS = amyothrophic lateral sclerosis; CLP1 = cleavage factor polyribonucleotide kinase subunit 1; FTD = frontotemporal dementia; mt = mitochondrial; MS = multiple sclerosis; Nsun2 = NOP2/Sun RNA methyl transferase 2; PCH = pontocerebellar hypoplasia; PD = Parkinson’s disease; PGRN = progranulin; PKM2 = pyruvate kinase M2; tsRNA = tRNA-derived small RNA; sncRNAs = small non-coding RNAs.

^a^Compare to the reports from Sanadgol *et al*.^51^ and Elkordy *et al*.^52^

### Widened horizons via functional variety

At the molecular level, proposed tsRNAs functions include many modes, whose details exceed the focus of this article and are covered in a number of comprehensive reviews.^19,39,79,80^ Briefly, tsRNAs may function by: (i) limiting translatability of complementary mRNAs in a miRNA-like manner^81,82^; (ii) impacting mRNA transcripts by interacting with RNA binding proteins (RBPs) and decreasing transcript levels,^83^ or inversely increasing transcript levels by facilitating its stability^84^; (iii) interfering with the translation machinery, leading to inhibition of translation initiation,^63,85^ regulating ribosome biogenesis and facilitating translation^86^; (iv) controlling histone levels and regulating the production of non-coding RNAs^87^; and (v) regulating transposable elements^88^ or other, as yet unknown roles.

As the above indicates, the mechanisms of tsRNAs function are far more complex and diverse than those of other small non-coding RNA families, such as miRNAs. Importantly, several tsRNAs differ only slightly from miRNAs in their sequence, but the great majority of tsRNAs show more diversified length and cell-type variability. However, this did not prevent the early misidentification of several tsRNAs as miRNAs. Their tsRNAs identity was only discovered several years later,^89^ followed by their removal from the miRNA directory. While both miRNAs and tsRNAs may lead to translational repression based on complementarity with specific mRNA targets, the variable length and possible structural conformations of tsRNAs and their chemical modifications (refer to the ‘Challenges and opportunities in tsRNAs research’ section) indicate diverse interactions with mRNAs and other targets. Furthermore, while tsRNAs are a unique and diverse family of small non-coding RNAs, it seems that they may also share some similarities with piRNAs (PIWI-interacting RNAs). piRNAs are usually 24–31 nucleotides (nt) long (which is also true for some tsRNAs) and are best known for protecting the germline by suppression of transposable elements.^90^ At least one report identified a somatic tRNA-derived piRNA in cancer cells,^91^ although other authors argue that most of the piRNAs and other small non-coding RNA families overlapping sequences are probably wrongly annotated to the piRNA databases, as their biogenesis does not involve the PIWI pathway.^92^

Importantly, the non-miRNA-like roles of tsRNAs seem to be particularly complex and depend on many yet unknown facts. Identifying the mechanisms of tsRNAs function is therefore of utmost importance for future studies of their role in brain development, homeostasis and neurological disorders. Furthermore, a recent hypothesis by Hagey and co-workers,^93^ validated in COVID-19 immunization studies, argues that the structure of the molecules targeted by small RNAs may determine their interaction better than sequence complementarity. Viewing structural characteristics as the key elements driving the immunological potency of small RNAs^93^ established basic principles for understanding their role in immune response. These findings emphasize the importance of devoting special attention to the structural features of tsRNAs and tsRNAs-complementary regions. In this context, tsRNAs may also function as aptamers, whereby their structural properties determine their binding capacities to proteins or other targets.^94^ The aptamer mechanism of action is very simple, and it might be widely evolutionarily conserved across diverse life domains.^94^

### Acquiring novel activity modes

Assessing evolutionary principles, tRNA is an ancient molecule, which has already been present in archaea and bacteria, with some differences between the diverse life domains. For example, archaea tRNA genes include multiple introns and split tRNAs (up to three genes, each encoding one part of the tRNA molecule).^95^ Several fascinating theories attempted to explain the evolutionary process of tRNA origins, including its emergence from two or even three hairpin molecules.^96^ A recent report argued that slight modifications in tRNAs create molecules capable of self-replication,^97^ supporting the theory in which life began with RNA.^98^ Correspondingly, modern life kingdoms employ different tRNA processing pathways, suggesting evolutionarily specific usage of nucleases.^16^

While it seems that tsRNAs were already observed in cancer tissues in the 1970s,^99,100^ early reports regarding tsRNAs generation also involved *Escherichia coli*, whose infection with bacteriophage T4 induced the anticodon nuclease and subsequent tRNA fragmentation.^5,101^ The conserved structure and function of tRNAs raise an additional intriguing but related question: may tsRNAs serve as interdomain/interkingdom signalling molecules between bacteria and their hosts? A recent study supporting this idea reported that tsRNAs produced by commensal bacteria of the *Rhizobium* species induce certain phenotypes, such as root nodulation in host plants.^102^ However, the extent that bacteria-host communication via tsRNAs are relevant in mammals, including humans remains to be elucidated. One report showed that a tsRNA produced by *E. coli* and packaged in bacterial outer membrane vesicles upregulated MAP3K4 expression by competition with host miRNAs, when transferred to a human colonocyte cell line.^103^ To the best of our knowledge, the direct connection between a bacterial pathogen/commensal and host gene expression has not yet been shown in mammals. However, another fascinating link between bacteria and their host is the observation that the microbiome impacts the expression and modification profiles of tRNAs in the host.^104^ The microbiome is a collection of commensal microorganisms accompanying the host throughout their life, with the biggest community found in the gastrointestinal tract. Perturbations in the microbiome have been identified in several disease conditions, including CNS disorders.^105^ Intense research efforts have been invested in understanding this connection and elucidating whether the microbiome may be in any way involved in the pathogenesis of neurological disorders. In this context, the link between tRNA levels and commensal microorganisms provides an additional layer of information. It thus seems that tissues from specific pathogen free mice (harbouring conventional microbiome) show different tRNA levels than tissues collected from germ free mice.^104^ Germ free mice are not exposed to pathogens/associated with commensals throughout their life, providing an experimental model for studying the divergent influence of the microbiome on development, physiology and diseases of its host.^106^ Intriguingly, changes in the tRNA transcriptome of investigated animals were tissue-specific, showing marked alterations in the levels of brain tRNA transcripts.^104^

### Subcellular location-related activities

Owing to their critical role in translation, tRNAs should primarily be located in the cytoplasm, near the ribosomes; and the cleavage of those tRNAs would yield tsRNAs localization in the cytoplasmic domain. However, neurons have quite complex cellular architecture, including numerous dendrites and extended axons, whose structure may change with age or in response to insults, disease or psychological stress.^107^ Furthermore, experimental evidence has shown that translation processes also take place near synapses.^108^ Spatial transcriptomics may reveal whether and which particular tsRNAs reach those extremities, and establish weather their levels change with age, under acute stress or with emerging disease, as seems to be the case for miRNAs^109^ and in some disorders of the CNS.^110^ While tsRNAs cellular localization studies have not yet been done on neuronal cells, a recent report investigating subcellular localizations of small non-coding RNAs, including tsRNAs, in three breast cancer cell lines highlighted preferential compartments for specific molecules, even for transcripts differing only by few nucleotides in sequence. This may also be linked with unique functions within a specific subcellular compartment.^111^

### Shuttling and delivery messengers

In addition to the roles of tsRNAs in intracellular function, small non-coding RNAs may act as messenger molecules that can be shuttled between cells and throughout the body.^112^ Cell-free small RNAs, including tsRNAs,^113^ are transported in extracellular vesicles or associated with carriers, such as ribonucleoproteins or lipoproteins.^114^ However, the potential of neuronally produced and vesicles-packed tsRNAs as mediators in brain-body communication has not yet been explored in an in-depth manner. One study profiled the content of extracellular vesicles including small non-coding RNAs, from several brain regions of one human donor (corpus callosum, orbital/frontal cortex, postcentral gyrus, occipital gyrus, thalamus, hippocampus, medulla and cerebellum) and showed that the reads mapping to tRNA were particularly enriched in vesicles derived from the medulla.^115^ Theoretically, both axons and released extracellular vesicles of neurons may harbour tsRNAs,^116^ which would be pivotal for tsRNAs playing a role in inter-neuronal communication. Specifically, 5′-tsRNAs were identified in cholinergic synaptic vesicles.^64^ Moreover, extracellular tsRNAs seem to differ from the intracellular ones and reflect cellular stress more accurately than miRNAs.^117^ Furthermore, tsRNAs are particularly enriched in biological fluids, including the CSF,^118^ which may shed some light on their perturbation in brain diseases and calls for identifying which cell types they originated from.

## Challenges and opportunities in tsRNAs research

tsRNAs differ in many ways from other members of the small non-coding RNA family. One difference is the extensive chemical modifications of their parent molecules in specific nucleotide positions, which are essential for tRNA function in protein synthesis.^16^ There are 39 types of modifications in human tRNAs encoded in the nucleus and 18 modifications in mitochondrially encoded tRNAs.^119^ Specifically, eukaryotic tRNAs contain on average 13 modifications.^120^ Also, tsRNAs may carry chemical modifications inherited from their originating tRNAs,^119,121^ and tRNA modifications control tsRNAs production (for reviews see Lyons *et al*.^16^ and Akiyama and Ivanov^122^), which may either protect the parent tRNA molecule from cleavage^123^ or facilitate tsRNA generation (as shown in yeast cells^124^). The tRNA modifications are also tissue-specific, but undergo changes depending on the cellular states. Hypoxia, for example, was shown to impact the modification status of tRNAs,^125^ and activation of T cells has been shown to change the tRNA modification profiles.^126^

Deficits interfering with chemical tRNA modifications lead to ‘tRNA modopathies’, which include several inherited diseases affecting nervous system functioning.^127^ These disorders predictably involve errors in translation, but also impact the generation of tsRNAs, as in the case of NSUN2 defects. NSUN2 (NOP2/Sun RNA methyl transferase 2), a member of the methyltransferase family, the largest group of tRNA modifiers,^128^ catalyses cytosine methylation (m^5^C) in specific tRNAs, and this modification inhibits tRNA cleavage by angiogenin.^129^ Correspondingly, mice with Nsun2 knockout show increased accumulation of 5′-tsRNAs linked to accelerated neuronal death,^65^ as well as defects in neural stem cells,^66^ highlighting again the risks involved in excessive 5′-tsRNAs accumulation. Interestingly, the NSUN2 effects seem to be counteracted by actions of TET2 (ten-eleven translocation 2 protein), previously known for oxidizing 5-methylcytosine in DNA. Recent reports show that TET2 can bind tRNA and that hydroxymethylation of cytosine also affects tsRNA production. Correspondingly, Tet2 knockout in mice leads to decreased 5′-tsRNAs and increased 3′-tsRNAs levels.^130^

Mutations in another tRNA-modifying enzyme, TRMT10 (tRNA methyltransferase 10) lead to pathogenic phenotypes associated with microcephaly, intellectual disability and diabetes.^131^ Interestingly, experiments with pancreatic beta cells showed that TRMT10 defects are linked to increased apoptosis due to accumulation of tRNA-Gln-derived 5′-tsRNAs.^131^ However, Trmt10a knockout murine brains did not show upregulation of these tsRNAs,^132^ which raises additional questions about tissue specificity of tsRNAs abundance and actions. Another methyltransferase, TRMT1 (tRNA methyltransferase 1) has been shown to be cleaved by the SARS-Cov2 protease, leading to declined TRMT1-mediated modifications in case of SARS-Cov2 infection,^133^ once again underscoring the changeable character of tRNA modifications.

Numerous tRNA modifying enzymes have been characterized in recent years, and the activity modes of 23% of the genes involved in tRNA modifications await in-depth discovery.^128^ Apart from their impact on tsRNAs abundance, the modification profiles may contribute to tsRNAs interactions with their targets,^134^ thus altering their biological activities. Together, these features may shed new light on specific genetic brain disorders^135^ (e.g. NSUN2^136^ or CLP1 affecting tRNA processing and functions^57,137,138^). Compatible with the above, tRNA mutations might affect both the chemical modifications of tsRNAs and their impact on tsRNA activities. Those may involve interrupted recognition of the complementary mRNA targets of the affected tsRNAs, altered capacities of tsRNAs to interact with specific RNA-binding proteins, changed ability to accumulate in stress granules, all of these options together or other, yet unknown functions.

**Figure 2:**
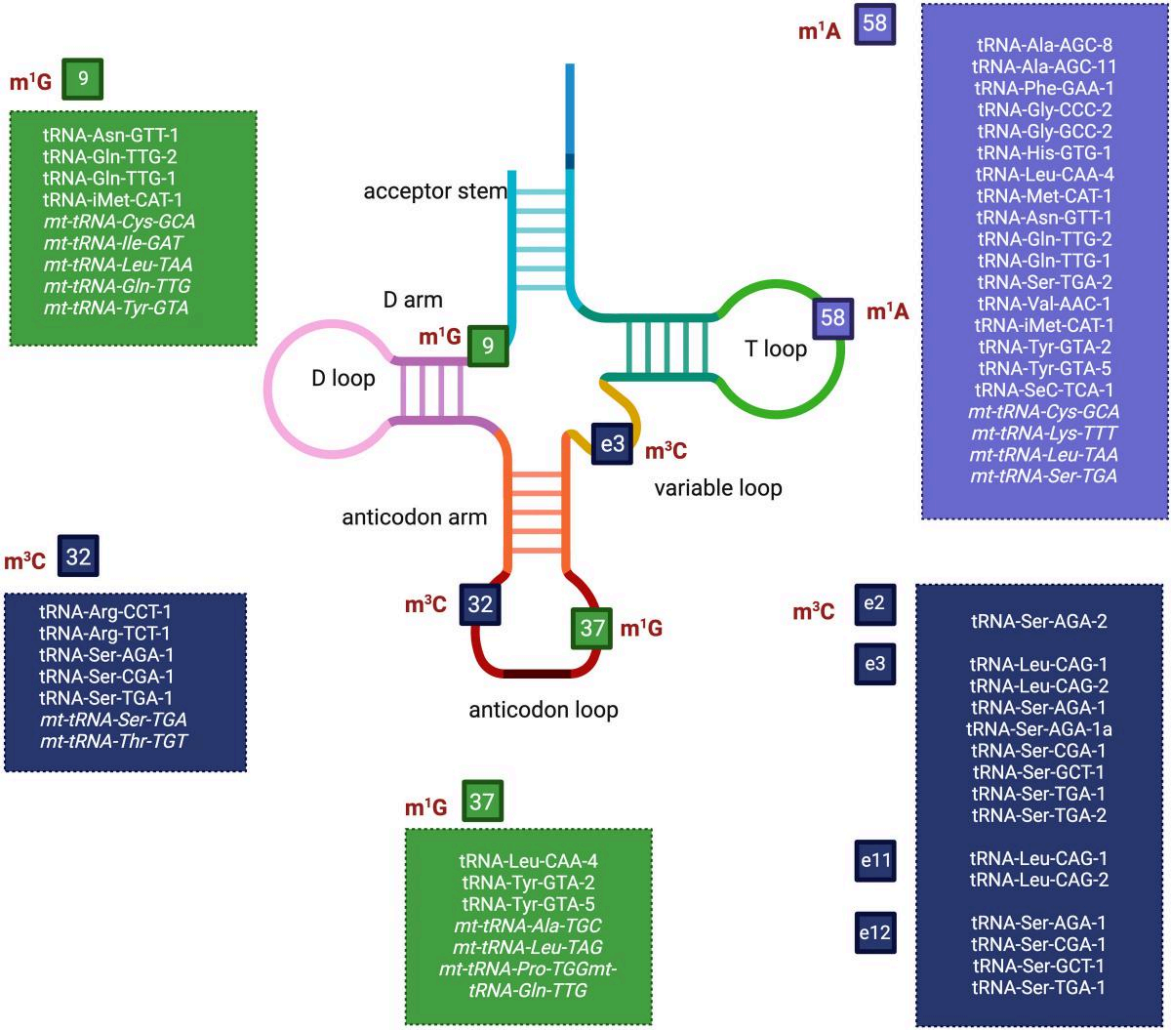
**tRNA modifications lead to difficulties in reverse transcription during cDNA synthesis.** Shown are the most problematic tRNA modifications, their positions in the tRNA molecule^139^ and human tRNAs decorated with these modifications according to Lei *et al*.^140^ Mitochondrial tRNAs are shown in italics. Created in BioRender. Winek, K. (2025) https://BioRender.com/ld1wasc.

**Figure 3:**
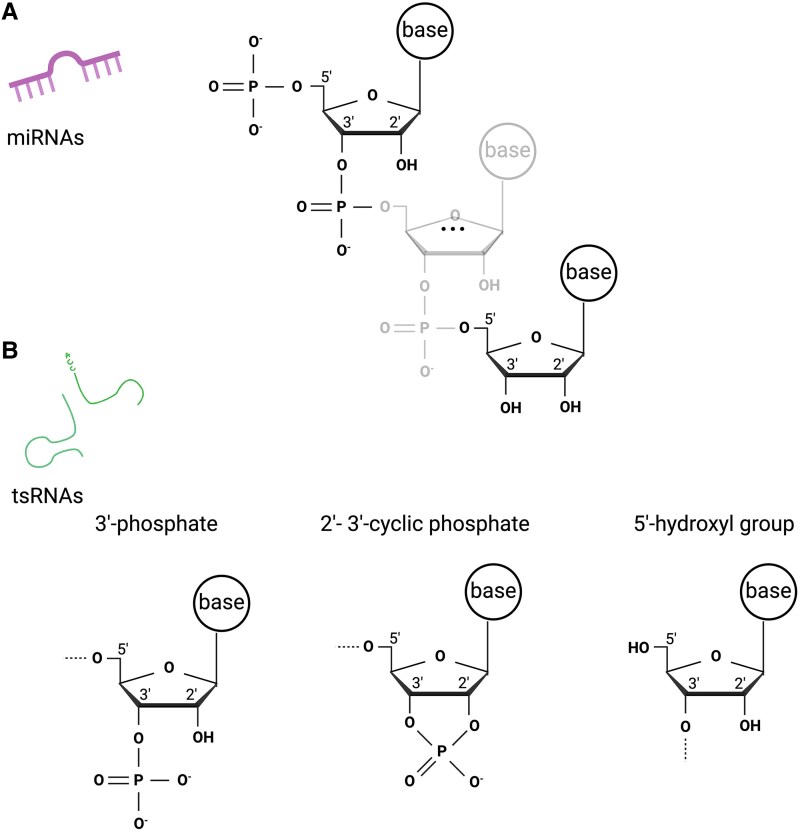
**Unconventional termini lead to problems with adapter ligation during library preparation.** (**A**) While small RNAs, such as microRNAs, include a phosphate group on the 5′ end and a hydroxyl group on the 3′ end, the tRNA cleaving enzymes introduce unconventional modifications of tsRNAs ends, such as those shown in **B**.^80,94^ Created in BioRender. Winek, K. (2025) https://BioRender.com/b7uyyo5.

tsRNA modifications and altered termini also contribute to technical challenges in tsRNAs sequencing.^80^ Modifications such as m1A (N1-methyladenosine), m3C (N3-metylcytosine) and m1G (N1-methylguanosine) stop reverse transcriptase activity or cause incorporation of incorrect nucleotides^139^ (Fig. 2). Furthermore, tRNA-cleaving enzymes leave specific termini in produced tsRNAs, including a 5′-hydroyxyl group, a 3′-phospate or a 2′-3′-cyclic phosphate. These differ from the classical miRNA ends (5′-phosphate and 3′-hydroxyl group) and complicate adaptor ligation during standard library preparation (Fig. 3).^80,94^ Special techniques have been developed to address these issues, for example, DM-tRNA seq,^139^ PANDORA-seq,^141^ CPA-seq^142^ ARM-seq^143^ or mim-tRNAseq^144^ using thermostable group II intron reverse transcriptase (TGIRT) that can read through many modifications and/or enzymatic removal of tRNA modifications and introduction of typical termini^145^ (for a comprehensive review on tRNA sequencing methods, see Padhiar *et al*.^145^ and Zhang *et al*.^120^). Furthermore, advances in sequencing technologies, such as direct (without a PCR preamplification step) nanopore RNA sequencing may help to solve these problems.^146,147^ Recently, a new ligation-independent sequencing approach identified long 3′-tsRNAs with blocked 3′ ends, which remained undetected using conventional methods and revealed tissue-specific expression of these tsRNAs when comparing brain and testis.^148^ Interestingly, tRNA modifications, aminoacylation and fragmentation appear to be inter-coordinated, as identified by Hernandez-Alias *et al*.^149^ Additionally, in their study, the brain significantly differed from other analysed tissues (muscle, heart and liver) in its m^1^A58 modification (Fig. 2).

Another complexity in tsRNA research involves data processing and analysis pipelines and the use of different classifications and naming systems, as mentioned before (refer to ‘The basics of tsRNAs: a beginner's guide’ section). Cabrelle *et al*.^150^ summarizes some of the currently available tools for sequencing analysis and target predictions. This list, however, expands quickly and novel pipelines are continuously being made available. Additionally, tRNA sequences pose a challenge when it comes to correct annotation, because of multiple copies of tRNA genes in the genome and mismatches generated during sequencing due to modifications.^151^ Sequence similarities in parent tRNA molecules, for example isodecoders, which diverge in some cases by only one nucleotide in position other than the anticodon,^144^ will of course also affect tsRNAs, causing problems in correctly mapping these molecules.

## Transfer RNA fragments in the healthy and diseased brain

**Figure 4:**
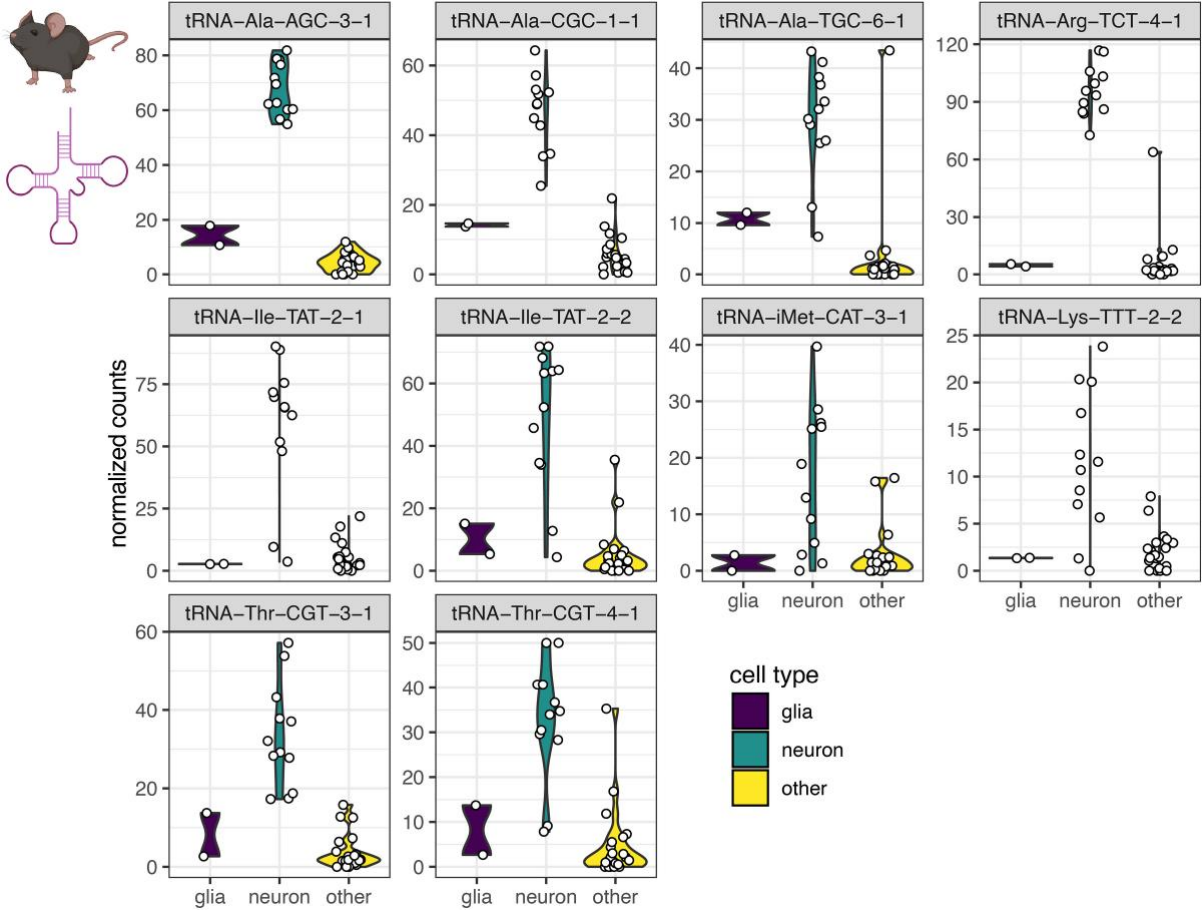
**tRNA gene usage in adult murine brains based on ATAC seq.** ^154^ Shown are the top 10 enriched tRNA genes with at least 2-fold higher transcript levels in neurons compared to all other cell types, from the dataset of Gao *et al*.^154^ Created using R Studio version 2023.03.1+446 and BioRender. Winek, K. (2025) https://BioRender.com/3k0ve55.

tRNA mutations play diverse roles in numerous human diseases, including neurological disorders (reviewed by Burgess and Storkebaum^35^ and Schaffer *et al*.^152^). Accumulating knowledge has underscored the importance of tRNA expression patterns in the CNS, and as tRNA transcript availability determines tsRNAs production, it will be important to understand the role of tsRNAs in the maintenance of physiological steady state.^31,153^ Recent studies demonstrate that nervous system tRNAs may be divided into brain, neuronal or neuronal-type specific ones (Fig. 4).^31,154^ In this context, several datasets from the brain and cell-type specific populations revealed distinct expression levels of isoacceptor tRNAs^10,31^ and therefore indicated that tsRNAs may have both ubiquitous as well as cell type-specific expression patterns. Correspondingly, human and mouse neurons are both particularly enriched in various tRNAs with alanine anticodons,^154^ and compared to seven other tissues, the human brain shows the highest levels of tRNA transcripts of mitochondrial origin.^155^ In contradistinction, in mice, the heart shows the highest levels of mitochondrially-encoded tRNAs among several tissues.^156^ In a mouse model, loss of only one neuronal tRNA, nTr20 [tRNA-Arg (TCT), Fig. 4, top right], alters translation and neurotransmission and increases resistance to seizures.^157^ Inversely, mutated tRNA-Arg (TCT) is linked to neurodegeneration.^158,159^ A very recent report using conditional Pol III knock-in mice (polymerase III responsible for tRNA gene transcription) and ChIP-seq (chromatin immunoprecipitation sequencing) showed specific expression patterns of neuronal versus non-neuronal tRNAs.^31^ The authors identified 40 tRNA genes whose expression was upregulated in neurons as compared to glia and 19 tRNA genes with higher expression levels in glial cells. Further, similar to Gao *et al*.^154^ they showed high neuronal expression of tRNA-Arg-TCT-4-1, tRNA-Ile-TAT-2-1 and tRNA-Ile-TAT-2-2. tRNA-Arg-TCT-1-1 showed particular expression in glial cells and marked differences between neuronal subpopulations. Among investigated neurons (Purkinje cells, dopaminergic neurons, layer V cortical neurons, spinal cord cholinergic neurons, cerebellar and dentate gyrus granule cells and inhibitory interneurons), cerebellar granule cells were very distinct in their tRNA expression profiles.^31^ However, despite the increasing body of evidence about tissue and cell-specific tRNA distribution, our knowledge about the specificity of tsRNAs is still limited. For example, the mouse brain exhibits specific small non-coding RNA profiles compared to other organs.^160^ In comparison, the primate hippocampus is enriched in 5′-tRNA halves,^161^ but the swine hippocampus and amygdala show increased abundance of 3′-tRNA halves when compared to other tsRNAs.^162^ Furthermore, tsRNAs are enriched in the murine hippocampus and substantia nigra when compared to the spinal cord, where miRNAs seem to be the most prominent small non-coding RNA family.^78^

Generating a robust set of tsRNA expression patterns in the CNS, including different regions and cell types, would be of great interest to the scientific community interested in further advancing tsRNAs research.

### The potential of tsRNAs as (not only) biomarkers?

The brain is isolated from the blood by the blood–brain barrier (BBB) formed by endothelial cells, astrocytic end-feet and pericytes. CSF surrounding the brain, although produced by plasma filtration, is also separated from the blood by the blood–CSF barrier at the interface of choroid plexus epithelial cells.^163^ BBB permeability is increased in conditions such as ageing or CNS disease^164^ and CSF provides some information about the brain health and pathological changes, correspondingly offering diagnostic possibilities. The emerging concept that tsRNAs may control or fine-tune the activities of particular brain cell types further predicts altered tsRNAs levels not only in these cells, but also, in case of secreted tsRNAs—in blood or CSF, in CNS disorders. An early indication of the relevance of tsRNAs in nervous system diseases was the fact that the blood levels of certain tsRNAs were found to increase several hours prior to seizures in patients with chronic epilepsy.^67^ Despite the presence of tsRNAs in brain tissue and primary neuronal cell cultures, it is still uncertain if the elevated levels of prognostic plasma tsRNAs actually reflect release of those molecules from the CNS.^67^ While no mechanistic implication exists for tsRNAs role in epileptic activity, tsRNAs are known to be secreted from neurons.^67^

A further indication of the value of tsRNAs as biomarkers, was a study of patients with ALS that showed association of 5′-tsRNAs derived from tRNA-Val with a decreased rate of disease progression.^68^ The neuroimmune capacities of tsRNAs raise another issue of medical significance, as blood cells from patients suffering an ischaemic stroke showed parallel increases in diverse tsRNAs and concomitant decreases in the miRNA levels, with particular enrichment in molecules predicted to target cholinergic transcripts.^69^ Notably, stroke patients suffer increased risk of infections, which is facilitated by the brain-injury triggered suppression of peripheral immune functions.^165^ Therefore, given the cholinergic control of inflammation,^166^ tsRNAs may be involved in orchestrating immune responses after stroke. Another study showed that tsRNAs elevation in the plasma of stroke patients is associated with the stroke outcome.^70^ Interestingly, expression patterns of tsRNAs (but not microRNAs) exhibited inverse changes in cellular compartments of blood and cells found in CSF in multiple sclerosis patients. In addition, expression levels of several tsRNAs in CSF cells showed differences between relapse and remission phases.^71^ Altered tsRNA profiles were also found in the blood and CSF of PD^72^  ^,73^ and Alzheimer's disease (AD)^74,75^ patients.

Profiles of small RNAs in the brain show differentially regulated tsRNAs in low-grade gliomas (benign tumours) and glioblastoma (malignant tumour of the CNS with very poor prognosis)^167^ that may be functionally significant. For example, NOTCH2 is a known promoter of glioma growth and recently, a 3′-tsRNA derived from alanine-carrying tRNA (termed by the authors CAT1—‘cancer associated tsRNA 1’,) has been identified as a regulator of NOTCH2 signalling.^84^ Notably, CAT1 inhibits the degradation of NOTCH2 transcripts by binding to a specific RNA-binding protein with multiple splicing capacities (RBPMS), which facilitates tumour growth.^84^ Interference with the production of CAT1 or the tsRNA itself may therefore offer a therapeutic option and calls for further studies.

AD patients had altered profiles and expression levels of cortical small non-coding RNAs and specific chemical modification patterns, as well as decreased levels of longer tsRNAs (30–40 nt) derived from tRNA-Tyr and tRNA-Arg,^76^ The functional significance of these changes is still unclear. Finally, altered levels of mitochondrial-originated tsRNAs predicted to target cholinergic mRNAs decline with age and disease in brain regions associated with higher cholinergic activity, such as the nucleus accumbens. This was observed in females, but not males^77^ and specifically correlated with cognitive, rather than the neuropathological AD changes. It might suggest that age-dependent loss of mitochondrial genome-originated tsRNAs may functionally contribute to impaired cognition, which calls for additional investigations.^63^ Further studies will be required to find out if the ageing-related cognitive decline of cholinergic activities in males and females under chronic anti-cholinergic medications^168^ is likewise linked to mitochondrial-originated tsRNAs targeted to cholinergic transcripts, and if it is more pronounced in females.

In mouse models of neurodegenerative diseases (ALS, FTD and PD), tsRNAs are dysregulated, with upregulation of specific 5′-tsRNAs produced by angiogenin in ALS models and downregulation of 3′-tsRNAs produced by Dicer in the TDP43^A315T^ ALS and the Tau^P301S^ FTD models. Interestingly, one fragment, 3′-tsRNA from tRNA-Cys-GCA (also known as tRF-3003a) showed lower levels in Tau^P301S^ mice as compared to wild-type control animals. Additionally, this tsRNA was shown to operate in a miRNA-like manner, targeting, among others, transcripts related to synaptic activity.^78^

In summary, the fact that tsRNAs show specific changes in epilepsy, AD, ALS, Huntington’s disease (HD), FTD, PD, pontocerebellar hypoplasia and ischaemic stroke may reflect their capacity to mediate local effects in the CNS on the one hand, while also highlighting their involvement in brain-body communication on the other hand.^169^ In addition, tsRNAs may be involved in ageing-associated phenomena awaiting therapeutic intervention.^170,171^ One notable example refers to the previously mentioned ageing-related generation of tsRNAs leading to impaired glutamate synthesis with consequent impact on neuronal mitochondria.^63^ Also, tRNA production, processing and specific chemical modifications (e.g. pseudouridylation) have all been linked with ageing and may relate to lifespan regulation.^172^ Specifically, tsRNAs recruitment may be impaired by their age-related depletion from the brain following ageing and/or death of the originating neurons^60^ and mitochondria.^173^ Such processes may accelerate mitochondrial disintegration and disease development. Additionally, the general sex-related differences^174^ and the long-recognized mitochondrial damage in PD patients^175^ may underlie the altered tsRNAs profiles in their blood and CSF.^72^ For all of these reasons, clarifying the impact of tsRNAs on neuronal activities, brain function, and brain-body interactions may elucidate their roles as essential regulators of homeostasis and foresee future development of tsRNAs in diagnostics/therapeutics avenues.

## Brain tsRNAs: the Phoenix small RNAs

**Figure 5:**
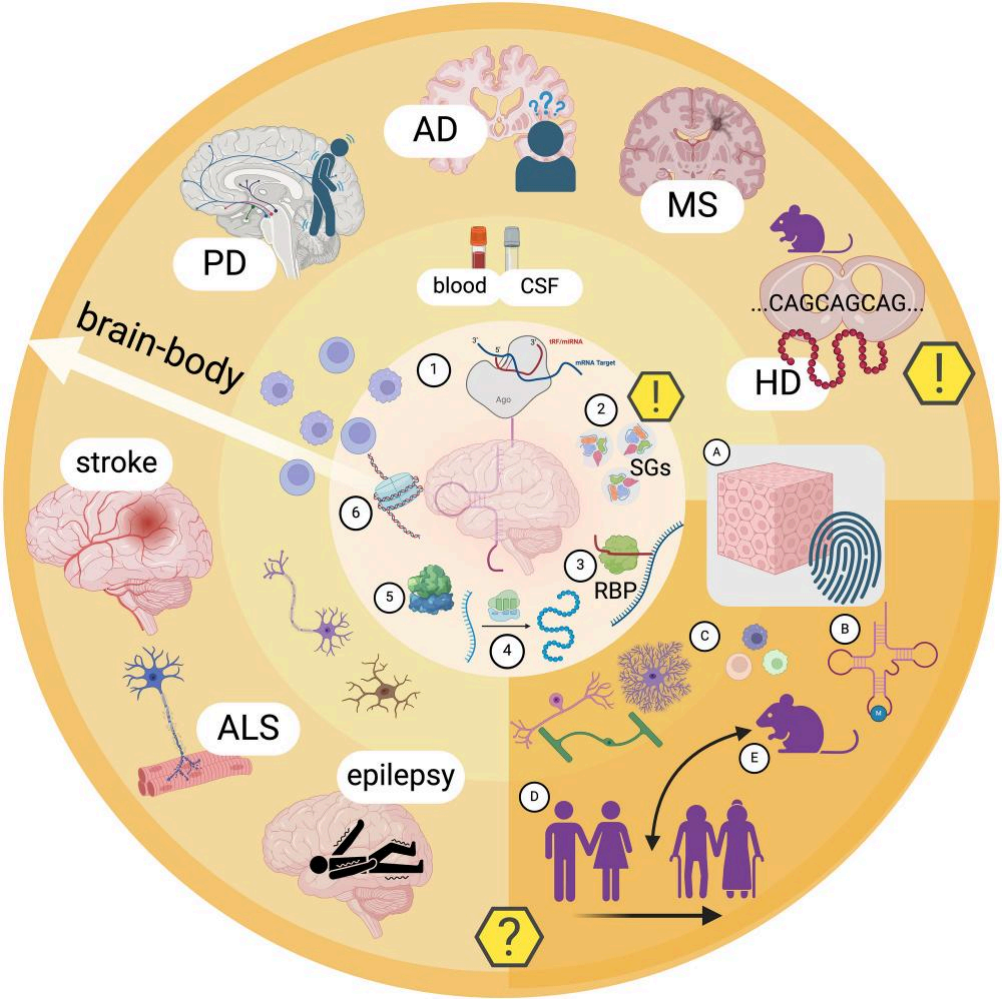
**Summary of tsRNAs functions, relevance in neurological disease and future questions.**  *Inner circle*: Examples of diverse tRNA-derived small RNAs (tsRNAs) functions: (1) miRNA-like mode of action and association with Ago proteins; (2) assembly of stress granules; (3) coupling with RNA binding proteins, further affecting mRNA transcripts; (4) modulation of the translation machinery; (5) facilitation of ribosome assembly; and (6) regulation of histone levels. tsRNAs are found in CNS cells, but also biofluids such as blood and CSF and their cellular compartments, increasing biomarker potential. *Bottom right quadrant*: challenges and open questions: (A) identification of tsRNAs signatures in tissues; (B) roles of tRNA modifications; (C) expression in specific cell types; (D) differences in tsRNAs expression between sexes and along age; and (E) interspecies differences. *Outer circle*: Examples of neurological disorders where tsRNA changes have been identified. AD = Alzheimer's disease; ALS = amyotrophic lateral sclerosis; HD = Huntington's disease; MS = multiple sclerosis; PD = Parkinson's disease (refer to the text for more explanations). Created in BioRender. Winek, K. (2025) https://BioRender.com/we6nj2e.

In conclusion, brain tsRNAs are a new and exciting family of small non-coding RNAs, which may have novel mechanisms of action, and are already raising a plethora of open questions.^89^ The presence of tsRNAs in the brain, especially in neurons and their potential roles in inherited and acquired neurological disorders as disease-specific predictive biomarkers and possibly as therapeutic targets, urgently warrants further study (for a summary of tsRNAs roles, molecular mechanisms and open issues, see Fig. 5). Once again, we have long overlooked a large family of biologically active molecules that were hidden in plain sight, derived in the non-random ‘act of recreation’^121^ from tRNAs and their precursor molecules. This somewhat recalls the history of discoveries linked with the non-coding genome, previously termed ‘junk DNA’.^176^ Nature allows no ‘junk’; and the tsRNAs intriguing family of nucleic acids clearly shows that the concept of superfluous molecules and structures is rather limited, especially in the brain.
